# Assessment of Drug-Induced QTc Prolongation in Mental Health Practice: Validation of an Evidence-Based Algorithm

**DOI:** 10.2147/RMHP.S426591

**Published:** 2023-10-13

**Authors:** Monica Zolezzi, Athar Elhakim, Iman A Qubaiah, Doaa Elsayed Mahmoud, Shorouq Homs, Waad Elamin, Engy Sherif Tawfik, Oraib Abdallah, Yassin Eltorki, Noriya Al-Khuzaei

**Affiliations:** 1 College of Pharmacy, QU Health, Qatar University, Doha, Qatar; 2 College of Health Sciences, University of Doha for Science and Technology, Doha, Qatar; 3 Pharmacy Department, Mental Health Services, Hamad Medical Corporation, Doha, Qatar

**Keywords:** drug-induced arrhythmias, QTc interval prolongation, algorithm, mental health practice, clinical decision tool

## Abstract

**Background:**

Drug-induced QTc interval prolongation (QTcIP) can lead to serious consequences and is often a concern for mental health practitioners, as access to experts such as cardiologists, for consultation is time-limiting and can delay treatment decisions. This research aimed at validating the content of an algorithm for the assessment, management and monitoring of drug-induced QTcIP in mental health practice.

**Methods:**

Following an initial face validity by content experts, a cross-sectional survey of mental health care practitioners with a 4-point Likert-type scale was used to assess the validity of the decision steps on the QTcIP algorithm (QTcIPA) by estimating the content validity index (CVI) and the modified kappa statistic (κ*). Participants’ open-ended comments were also thematically analyzed.

**Results:**

Mental health practitioners found the QTcIPA to be appropriate, safe, and evidence-based, as indicated by the high individual item CVI scores ranging from 0.89 to 1 for all of the steps/decision statements in the three domains assessed: appropriateness, safety and reliability of the references used. Five themes emerged from the qualitative analysis of the open-ended comments, of which three were identified as strengths, including practical usability, reliable references and beneficial for pharmacists. Two themes were recognized as limitations, namely, the need for additional clinical content and application barriers.

**Conclusion:**

These results suggest that the QTcIPA may be a useful tool for mental health clinicians at the time of prescribing medications with potential risk of QTcIP. Future research will explore the implementation of the QTcIPA into clinical practice using computerized decision support tools through web-based and mobile applications.

## Introduction

People with serious mental illness (SMI) such as those with schizophrenia, bipolar disorder and major depression, have significantly shorter life expectancy when compared to the general population.^1^ Although many factors contribute to this shorter lifespan, cardiovascular disease is the major culprit of the higher mortality observed among people with SMI.^1–3^ Although ischemic heart disease and related risk factors account for the majority of sudden cardiac deaths (SCDs) in this population, approximately 10% are unexplained and may be the result of cardiac arrhythmias.^4^,^5^

A number of psychotropic medications are known to prolong the corrected QT (QTc) interval.^6^,^7^ Prolonged QTc interval (QTcI) is associated with a condition called torsades de pointes (TdP), which is a life-threatening ventricular tachyarrhythmia.^8^ In people with SMI, these medications are also often used in combination, or with other non-psychotropic medications that have been associated with prolonging the QTcI, posing this population at an increased risk of TdP and SCD.^2^,^6^,^8^ In addition to medications, other risk factors associated with TdP should also be considered when assessing and monitoring drug-induced QTcI prolongation (QTcIP), such as age, gender, gene polymorphisms, hypokalemia and hypomagnesemia.^9^

Studies have demonstrated that clinicians often encounter challenges identifying TdP risk factors or medications that can can cause QTcIP at the time of prescribing.^10^,^11^ Because of an ever-increasing number of medications available and other clinical factors that must be accounted for during risk assessment, clinicians may face difficulties on how to evaluate, manage, monitor or refer patients at risk of QTcIP. Several algorithms have been developed to address these challenges at the point of prescribing and aid health care professionals with clinical decision making.^12–14^

We have previously reported on the development of a stepped-based algorithm specifically to assess the risk of drug-induced QTcIP in the psychiatric population,^15^ and reported the results of the evaluation of the algorithm’s content validity as assessed by a panel of cardiologists.^16^ In this article we report on the evaluation of the same algorithm by mental health practitioners, in an effort to address current practice gaps in the assessment, management and monitoring of drug-induced QTcIP risk, in settings where cardiology consultations are limited or hard to reach.

## Methods

### Study Participants and Sampling

Using a purposive sampling strategy, mental health practitioners (pharmacists and physicians) from the Mental Health Services at Hamad Medical Corporation (HMC) in Qatar and members of the American Association of Psychiatric Pharmacists (AAPP) in the United States of America (USA) were invited to participate through email. A participant information sheet along with a consent form was provided electronically to all potential participants. Those who agreed to participate were given access to the algorithm (Appendix 1), an educational module that showed participants how to use the algorithm, and the link to the online survey.

### Educational Module

Articulate 360^®^ software, an online platform for e-Learning, was used to create an educational module designed to orient participants on the clinical decision steps in the algorithm. Interactive scenarios were also provided to help participants practice using the algorithm, so that they would later be able to assess its usefulness when completing the survey.

### Survey

**Figure 1:**
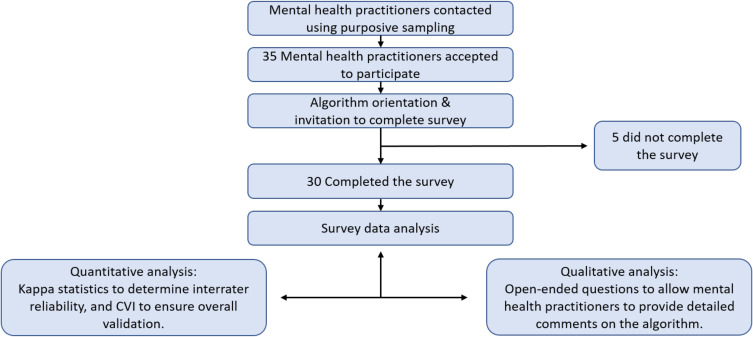
Schematic representation of the study process.

**Abbreviation**: CVI=Content Validity Index.

A self-administered survey containing quantitative and qualitative components was administered electronically via SurveyMonkey^®^. The link to the survey was included at the end of the educational module. The quantitative section of the survey consisted of three 4-point Likert scale sets of questions to identify participants’ viewpoints on the appropriateness, safety and reliability of the resources used in each step of the algorithm. The points were assigned as follows: 1 = not reliable, 2 = unable to assess reliability without revision, 3 = reliable but needs minor alteration, and 4 = very reliable. The qualitative section of the survey consisted of open-ended questions, to allow the participants to express their opinions, provide more specific feedback about the algorithm, and suggest modifications. Figure 1 provides a detailed outline of the study data collection process.

### Data Analysis

The ratings of mental health professionals of each algorithm step in terms of appropriateness, safety and reliability of references were collected through SurveyMonkey^®^ and were subsequently exported to an Excel^®^ data sheet for the analysis. The mean rating scores of each step were computed for each of the three attributes which were used to calculate the Item-Content Validity Index (I-CVI), a measure of the content validity of individual items in a scale.^17^ It was calculated using the following formula: I-CVI = (number of raters scoring an item with a 3 or 4)/(total number of raters).^18^ The I-CVI ranges from 0 to 1, and the cutoff for rejecting the item was established as a I-CVI <0.78. The average-CVI (Ave-CVI) was also calculated by adding the I-CVI scores and dividing them by the number of items.^17^ Ave-CVIs ≥ 0.9 are considered to reflect an excellent content validity.^17^

The modified Cohen’s kappa statistic (κ*) was also used to measure inter-rater reliability for categorical items.^19^ It is generally thought to be a more robust measure than simple percent agreement calculation, as κ* takes into account the possibility of the agreement occurring by chance.^19^ The coefficient was calculated using the following equation: ${\mathrm{k* = }}{{{\mathrm{I - CVI - PC}}} \over {{\mathrm{1 - PC}}}}$; where “PC” is the probability of random correlation coefficient, which is computed as follows: ${\mathrm{PC = }}\left[{{{\left[{{\mathrm{N!}}} \right]} \over {{\mathrm{A! }}\left({{\mathrm{N - A}}} \right)}}} \right]{\mathrm{X0}}{\mathrm{.}}{{\mathrm{5}}^{\mathrm{N}}}$; where “N” is the number of experts, and “A” is the number of raters agreeing on good relevance. As suggested by some authors, κ* values < 0 indicate no agreement, 0–0.20 as slight, 0.21–0.40 as fair, 0.41–0.60 as moderate, 0.61–0.80 as substantial, and 0.81–1 as almost perfect agreement.^20–22^

Qualitative content analysis of the open-ended survey responses was undertaken by one of the research team members (AE) using an inductive coding approach to code content into themes. This researcher then reviewed the responses with the preliminary themes and further defined them. The preliminary themes and their definitions were then shared with three additional researchers (IQ, DM, SH), who examined the data and determined whether themes needed to be added, modified or removed. After discussion amongst the researchers, discrepancies were resolved, agreement among researchers was reached, and a final hierarchical coding frame was developed.

## Results

As illustrated in Figure 1, a total of 35 mental health practitioners were contacted, of whom 30 completed the survey (18 from HMC and 12 from AAPP). The participants were predominantly females (60%), had a median age of 34.5 years, and their graduation years ranged from 1996 to 2019. The majority of the participants were pharmacists (86.7%) and the remaining were physicians (13.3%).

**Figure 2:**
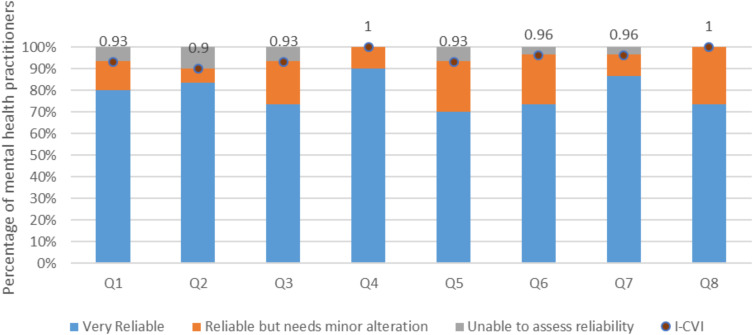
I-CVI scores for the appropriateness of algorithm decision steps.

**Notes**: Q1=Question 1: Assessing the drug using CredibleMeds^®^; Q2=Question 2: Assessing the drug dose, route, and drug interactions; Q3=Question 3: Assessing the risk by calculating the patient’s QTc prolongation score; Q4=Question 4: Assessing the need for ECG monitoring based on the QTc risk score; Q5=Question 5: Recommending therapy if the risk score ≤ 7 points; Q6=Question 6: Assessing baseline ECG if risk score ≥ 7 points; Q7=Question 7: If ECG shows QTc ≥ 500 ms, avoid therapy and consider cardiac consultation; Q8=Question 8; If ECG shows QTc ≤ 500 ms, recommend therapy with follow up ECG and physical exam at specified cases.

As presented in Table 1, and illustrated in Figure 2, mental health practitioners rated the appropriateness of the decision steps in the QTcIP algorithm (QTcIPA) with mean scores ranging from 3.68 to 3.83 out of 4. The appropriateness decision statement that had the highest overall average score (3.83) was in relation to avoiding therapy and considering cardiac consultation if ECG readings indicate QTcI ≥ 500 ms. Values of both the I-CVI and the κ* for the different appropriateness-related decision statements ranged from 0.93 to 1. No statements to assess the safety of the QTcIPA steps were rejected. The Ave-CVI for the appropriateness of the algorithm steps was 0.95.

**Table 1:** I-CVI Scores of the Decision Steps in the QTcI Prolongation Algorithm

	Minimum Mean Score (out of 4)	Maximum Mean Score (out of 4)	Average I–CVI (out of 1)
Appropriateness of the QTcIPA decision steps	3.68	3.83	0.95
Safety of the QTcIPA decision steps	3.7	3.83	0.95
Reliability of the references used in the QTcIPA decision steps	3.57	3.73	0.94

**Abbreviations**: QTcIPA, corrected QTc interval prolongation algorithm; I-CVI, item-level content validity index.

**Figure 3:**
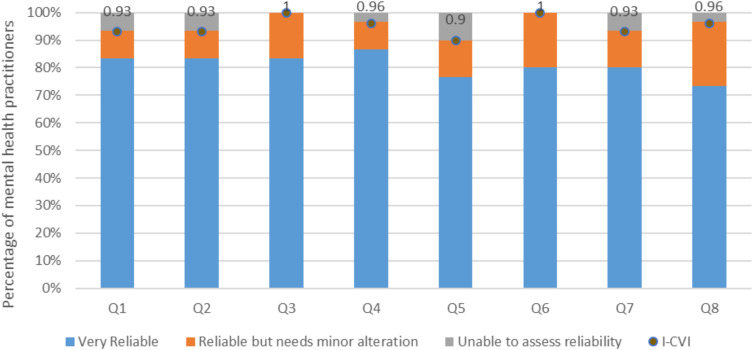
I-CVI scores for the safety of the QTc Prolongation Algorithm decision steps.

**Notes**: Q1=Question 1: Assessing the drug using CredibleMeds^®^; Q2=Question 2: Assessing the drug dose, route, and drug interactions; Q3=Question 3: Assessing the risk by calculating the patient’s QTc prolongation score; Q4=Question 4: Assessing the need for ECG monitoring based on the QTc risk score; Q5=Question 5: Recommending therapy if the risk score ≤ 7 points; Q6=Question 6: Assessing baseline ECG if risk score ≥ 7 points; Q7=Question 7: If ECG shows QTc ≥ 500 ms, avoid therapy and consider cardiac consultation; Q8=Question 8; If ECG shows QTc ≤ 500 ms, recommend therapy with follow up ECG and physical exam at specified cases.

As presented in Table 1, and illustrated in Figure 3, participants rated the safety of the decision steps in the QTcIPA with mean scores ranging from 3.70 to 3.83. The safety decision statement that was rated the highest (3.83) was in relation to assessing the need for ECG monitoring based on the QTcIP risk score. Values of both the I-CVI and the κ* for the different safety-related decision statements ranged from 0.9 to 1. No statements to assess the safety of the steps in the QTcIPA were rejected. The Ave-CVI for the safety of the algorithm steps was 0.95.

**Figure 4:**
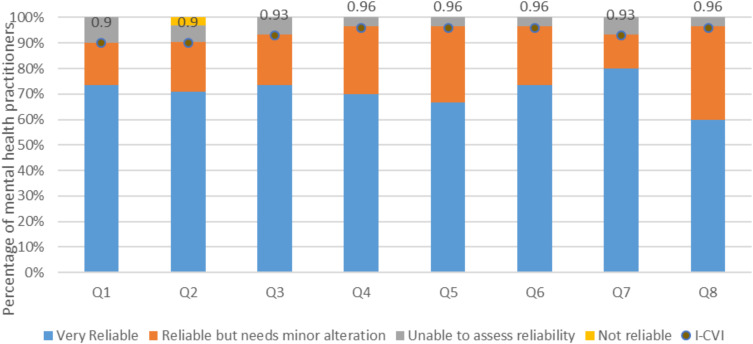
I-CVI scores for the reliability of references used in the QTc Prolongation Algorithm decision steps.

**Notes**: Q1=Question 1: Assessing the drug using CredibleMeds^®^; Q2=Question 2: Assessing the drug dose, route, and drug interactions; Q3=Question 3: Assessing the risk by calculating the patient’s QTc prolongation score; Q4=Question 4: Assessing the need for ECG monitoring based on the QTc risk score; Q5=Question 5: Recommending therapy if the risk score ≤ 7 points; Q6=Question 6: Assessing baseline ECG if risk score ≥ 7 points; Q7=Question 7: If ECG shows QTc ≥ 500 ms, avoid therapy and consider cardiac consultation; Q8=Question 8; If ECG shows QTc ≤ 500 ms, recommend therapy with follow up ECG and physical exam at specified cases.

As presented in Table 1, and illustrated in Figure 4, mental health practitioners rated the reliability of the references used in the different steps in the QTcIPA with mean scores ranging from 3.57 to 3.73. The appropriateness decision statement that had the highest overall average score (3.73) was in relation to avoiding therapy and considering cardiac consultation if ECG readings indicate QTcI ≥ 500ms. Values of both the I-CVI and the κ* for the different appropriateness-related decision statements ranged from 0.9 to 0.97. No items to assess the safety of the QTcIPA steps were rejected. The Ave-CVI for the appropriateness of the algorithm steps was 0.94.

Analysis of the Ave-CVI scores between the two groups of participants (from HMC and AAPP) showed slight differences. Overall, the Ave-CVI was slightly higher in the AAPP group of participants compared to the HMC group: 0.95 vs 0.94 for the appropriateness, 0.95 vs 0.94 for the safety, and 0.94 vs 0.92 for the reliability of references used, respectively. The AAPP participants gave the highest rating for the statement “Assessing the drug using CredibleMeds^®^” for the appropriateness, safety and reliability of the references used in the QTcIPA decision steps, as well as for the statement “Assessing the need for ECG monitoring based on the QTcIP risk score”, but only for the safety assessment of the QTcIPA decision step. The HMC participants gave the highest rating for the statement “Assessing the drug dose, route, and drug interactions” for both, the appropriateness and safety of the QTcIPA decision steps, as well as for the statement “Avoiding therapy and considering cardiac consultation if ECG readings indicate QTcI ≥ 500ms” for the reliability of the references used in the QTcIPA decision steps.

### Qualitative Assessment of the QTcI Prolongation Algorithm

**Figure 5:**
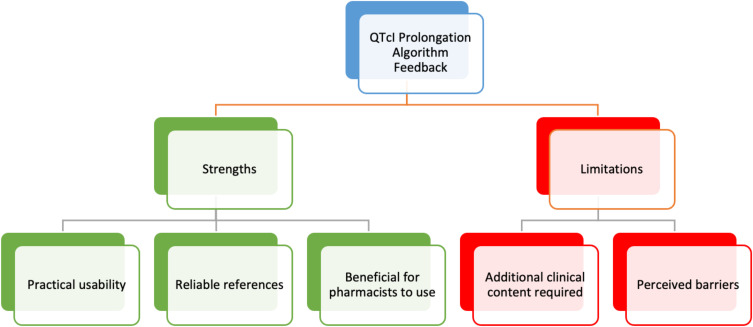
Emerging themes from participants’ feedback about the QTcI Prolongation Algorithm decision steps.

**Abbreviation**: QTcI, corrected QT interval.

As illustrated in Figure 5, there were a total of 5 themes that emerged from the participants’ feedback on the QTcIPA, which were organized using a hierarchical coding frame, identifying the algorithm’s strengths or limitations.

There were 3 emerging themes considered as strengths of the QTcIPA, these are described below along with supporting comments made by the mental health practitioners in the survey:

### Practical Usability

This theme consists of the participants’ feedback regarding the ease of using the algorithm. Some participants outlined that the algorithm steps were practical and preferred such an approach to assessing and managing QTcIP in practice, as the steps contained clear cut-off points and facilitated decision making.

It is finally an algorithm to plan QT based interactions management and also to manage patients with pre-existing QT interval prolongation. (HMC4)

Seems practical and helpful in daily clinical settings. (HMC10)

Provides clear-cut guidance individualized to patient risk. (AAPP1)

### Reliable References

This theme describes the participants’ views regarding the references utilized in the QTcIPA. The references were described as reliable and evidence-based, with most participants highlighting the use of the Tisdale Risk Score, a validated tool,^23^ and CredibleMeds^®^, an accessible online evidence-based resource.^24^

Using CredibleMeds^®^ database which is the standard reference for Drug-Induced TdP and QTc Interval Prolongation. Using Tisdale Risk Score for risk stratification which is already validated and can be accessed easily using multiple websites like MDCalc, etc.) (HMC11)

Crediblemeds® App is auite reliable. (HMC16)

Despite the references being described as reliable and evidence-based, some participants felt that the QTcIPA needed to incorporate additional reliable resources.

Using only credible meds as a source seems iffy. I would also use the lit search links they provide maybe as another safety check (AAPP7)

Relying on a website or application to determine safety of medications might not be considered best practice. References and scientific papers are a better and more reliable resource. I would feel more safe relying on a medication list generated by HMC pharmacy department with listed references. (HMC13)

### Beneficial for Pharmacists to Use

This theme emerged from comments supporting the use of the algorithm by pharmacists in practice. Several pharmacist participants felt comfortable and confident with using the QTcIPA in practice. Pharmacist use was also supported by other mental health practitioners.

I think pharmacists are well-positioned to monitor and manage QTc prolongation risk for psychiatric patients, and this could be a good resource to do that. (AAPP2)

It helps to make a more concrete decision based on facts and helps to organize contributing factors. (AAPP3)

This algorithm is reliable to use by pharmacist to discover any risk to patient for QTc prolongation before to avoid any serious risk to patient. (HMC3)

I believe it would be beneficial taking into consideration the nature and seriousness of the problem. (HMC10)

One pharmacist participant also identified the tool as a resource that could be utilized to support clinical recommendations made in practice.

I think it would be useful when explaining/justifying concerns over medication options and possible QTc prolongation. (AAPP9)

There were 2 emerging themes that were considered as limitations of the QTcIPA, these are described below:

### Additional Clinical Content Required

This theme describes emerging recommendations regarding the clinical content in the algorithm. Recommendations were made related to clarifications needed to avoid misinterpretation or misuse of the algorithm. These include adding a definition for a baseline ECG, how points are assigned to patients using the algorithm and identifying that the Tisdale Risk Score has not been studied in the outpatient population.

Clarify what a “baseline EKG” is or name it something else (i.e. current EKG). (AAPP8)

Tisdale Risk Score is only validated in admitted patients. (HMC11)

For the most part, the clinical content was deemed as a strength. However, recommendations emerged regarding certain clinical aspects that could be explained in more detail. These included: the different ECG cut-off points for males versus females, consideration of drug interactions, direction on which QT correction formula should be used, recommendations on modifying adjustable risk factors and on using alternative drugs from the same class, the addition of other important risk factors and the requirements to refer patients with a QTc > 500ms.

The algorithm does not account for different gender cut point of QTc 450 (males) vs. 470 for (females) (HMC16)

More clear/consistent guidance about when it is appropriate to change therapy to another agent in the same class with lower risk and what formula to use to calculate QTc. (AAPP2)

Consider looking into other QTc risk literature, other than just Tisdale, for expanded risk factors. (AAPP4)

Like everything else in psych, it feels very rigid for the patient population. As long as the user exercises clinical judgment and has a good grasp on patient care, it should be relatively safe. (AAPP5)

### Perceived Barriers

This theme represents comments that identified barriers or factors which may impact the implementation of the QTcIPA in practice. These barriers were described as resistance to change, whether it is due to health care professional/physician resistance with accepting recommendations, completing algorithm training and agreeing on QTc prolongation parameters, patient resistance with changing medications and cooperating during assessments, or institution resistance with approving and disseminating the algorithm for use.

Physicians or patients may not accept the alternative medication option if the QTc prolongation risk is high. Patients may refuse frequent ECG monitoring. (HMC5)

Accessing [the algorithm], some disagreement about QTc prolongation parameters, one of my colleagues uses much more conservative criteria. (AAPP1)

Physician resistance, time, and institutional protocols. (AAPP5)

Furthermore, barriers related to feasibility of use emerged. In contrast to the “practical usability” theme, some participants viewed the algorithm design as complex and faced issues utilizing it. These issues were mainly related to the multiple steps, calculating the risk score and accessing an external website which requires internet access and registration. Another participant supported the use of the algorithm. However, only after modifications are made.

Long algorithm, found it sometimes difficult to follow. (HMC6)

Slightly complicated, hard to grasp from the first time, needs to be looked at when assessing the patient. (HMC10)

[Weakness of the Algorithm is] the need to use an external website (HMC18)

I feel like this could be a bit complicated/cumbersome for many providers to work through on a patient-by-patient basis. (AAPP6)

I do not see any barriers if above alterations are considered. (HMC13)

Additional barriers related to feasibility included issues such as the ability to obtain ECGs, patient information and cardiac consultations, as well as the time needed to utilize and incorporate the algorithm into practice. Further supporting this theme is the recommendation for automation. Participants highlighted that automation is needed to minimize the risk of miscalculation of the points used in the algorithm and/or misinterpretation.

It has a lot of steps which may be confusing, and contains elements that may be vulnerable to miscalculation/misinterpretation. If it could be incorporated into an [electronic medical record] and automatically calculated as a clinical decision-making support that would be more user-friendly and safe. (AAPP2)

More time-consuming and subject to error if not incorporated into EMR. (AAPP2)

The time to go through this assessment on several patients would likely prevent widespread usage, but I could see it being useful for specific cases. (AAPP6)

See Appendix 2 (Supplementary Table 1 and 2) for additional information that relates to each of the emerging themes.

## Discussion

This study provides further evidence on the validity of the QTcIPA steps, and on its safe applicability in the psychiatric setting, as rated by mental health end users, such as psychiatrists and pharmacists. Overall, a positive inter-rater reliability was observed with Ave-CVI, I-CVI and κ* values well above the recommended cut-offs. Although several algorithms have been proposed for the assessment of QTcIP, this is the first which has been validated by cardiology experts,^16^ and now through this evaluation, by mental health clinicians. Over the past decade, physicians have become more aware of the potential risk of TdP with the use of psychotropic medications, but there is significant variability in practice and policy at the individual and institutional levels. The QTcIPA has shown promise in addressing the variability and practice gaps encountered in psychiatric settings or community mental health services where accessing cardiology consultations can be challenging. A notable strength of the QTcIPA is its alignment with the American Psychiatric Association Council on Consultation-Liaison Psychiatry’s Workgroup on QTc Prolongation and Psychotropic Medications,^25^ as it incorporates key clinical considerations for assessing QTcIP risk, as endorsed by the American College of Cardiology. Furthermore, to prevent patient harm, there is a need for effective tools in clinical practice. Hospital systems have successfully utilized the drug lists available at www.crediblemeds.org^24^ to identify patients at risk of QTcIP. Decision-support programs based on these lists have been developed, validated, and proven effective in reducing potentially dangerous prescriptions to patients with prolonged QTc intervals or those at risk of QTcIP.^26^

During the qualitative analysis, participant feedback on the QTcIPA yielded five themes. Three themes were identified as strengths: practical usability, reliable references, and benefits for pharmacists, while two themes were recognized as limitations: the need for additional clinical content, and perceived barriers. While the QTcIPA demonstrates significant potential for positive outcomes, it is important to acknowledge the limitations identified in our study. By considering these limitations, further refinement and improvements can be made to enhance the QTcIPA’s effectiveness and applicability. Reliable references emerged during analysis of the qualitative data as a strength of the QTcIPA, however, some participants identified concerns with the use of CredibleMeds^®^. This is likely the reason for a slightly lower rating score for the reliability of the references used (mean scores ranging from 3.57 to 3.73) in the quantitative analysis. It is important to highlight though, that CredibleMeds^®^ is a well-known and widely used resource that provides information on the potential risk of QTcIP and TdP associated with various medications. It is a reliable database that categorizes medications into different risk categories based on their potential to prolong the QTcI. The use of CredibleMeds^®^ in clinical practice can be supportive for healthcare professionals in several ways. Healthcare providers and insurers are increasingly utilizing the lists of medications provided in this database as the foundation for clinical decision support systems and as metrics to assess the quality of prescribing. A scientific review committee regularly conducts thorough evaluations of the medications listed in CredibleMeds^®^ to assess their potential association with drug-induced QTcIP or TdP. Furthermore, an international advisory board, composed of world-known experts in drug safety and cardiovascular medicine, reviews the committee’s decisions and recommendations.^27^ Despite the concerns raised, CredibleMeds^®^ is a valuable, reliable resource that can be used in decision support tools. However, it is important to note that as with other tools, it is still essential for healthcare professionals to use clinical judgment and to consider individual patient factors when assessing the potential risk of QTcIP.

Some participants expressed that they found the QTcIPA steps to be practical and preferred using such an approach for assessing and managing QTcIP in practice. They appreciated the clear cutoff points and how the steps facilitated decision making. However, they also emphasized the importance of incorporating additional clinical content into the tool. Questions were raised specifically regarding the use of the Tisdale Risk Scoring Tool,^23^ which has only been validated in patients admitted to cardiac critical care units. This limits the generalizability of the tool to other patient groups and highlights the need for further evaluation of its use in the psychiatric setting. Although we believe that its incorporation in the QTcIPA offers more benefits to practitioners than limitations for its use in a hospital setting, it would be valuable to incorporate additional risk factors that were not considered in the Tisdale risk scoring tool, such as electrolyte abnormalities (eg, hypomagnesemia), bradycardia, ischemia, coronary spasm, thrombosis, structural heart disease, and gender.^28^,^29^

It was also highlighted that the QTcIPA may need more clarity on the ECG cutoff value and to consider modifying it to account for gender differences. Although some authors have presented strong evidence that links the QTcI of more than 500ms to an increased risk of arrhythmia,^30^ the exact association between the QTc and the risk of arrhythmias remains controversial. The evidence that suggests the risk is exponentially related to the extent of prolongation beyond normal limits (440ms for men; 470ms for women) is limited.^31^ Despite the uncertainty, as per the Maudsley Prescribing Guidelines, QTcIP remains an important measure to estimate the risk of arrhythmia and SCD, and proposed actions to manage patients with QTcIP who are taking antipsychotic medications, which are summarized in Table 2. Future incorporation of similar action plans should be considered for the QTcIPA.

**Table 2:** Management of QTc Prolongation in Patients Receiving Antipsychotic Drugs

QTcI value in miliseconds (ms)	Action	Refer to cardiology
<440 ms (men) or <470 ms (women)	No action required unless abnormal T-wave morphology	Consider cardiac review if in doubt
>440 ms (men) or >470 ms (women)	Consider reducing dose or switching to drug of lower effect	Consider
>500 ms	Stop suspected causative drug(s) and switch to drug with a lower effect	Immediate cardiology review is needed

Another comment raised by participants was in relation to the assessment of drug interactions while using the QTcIPA. Although Step 2 in the original algorithm development mentions the need to assess for drug interactions,^15^ it was pointed out that there was no clear distinction between pharmacokinetic and pharmacodynamic drug interactions. Further clarification is necessary to ensure consistent implementation of this step among users.

Despite several pharmacist participants expressing comfort and confidence in utilizing the QTcIPA in their practice, they also identified perceived barriers. These barriers primarily revolved around the time required to apply the algorithm and concerns regarding potential miscalculations or misinterpretations that may occur when used in practice. To overcome these barriers, the research team intends to automate the algorithm using a decision support tool, which will help streamline the process. However, it is important to note that clinical judgment remains essential, as with any other clinical decision support tools. Overall, the utilization of the QTcIPA in clinical practice was regarded as advantageous, garnering support for its implementation among pharmacists.

A study limitation identified was the small sample size and potential lack of representativeness. This may be due to the purposive sampling method adopted. To address this concern, our study included not only pharmacists, but also physicians who may encounter patients at risk of QTcIP. Additionally, our participant pool encompassed individuals from Qatar and North America, acknowledging the potential variations in clinical practice across different regions. This approach aimed to minimize bias and enhance the generalizability of our findings. While we acknowledge our intentions to achieve a larger sample size, it is worth highlighting that this study represents the second validation study conducted on the QTcIPA. In the first study, content was validated by field experts (cardiologists) while in this study, the perspectives of mental health practitioners complemented the results obtained in the initial content validation study.^16^

## Conclusion

This evidence-based QTcIPA underwent evaluation by mental health professionals, demonstrating a high degree of content validity in terms of its appropriateness, safety, and reliability in guiding decision-making processes. The subsequent stages involve refining the algorithm based on the feedback gathered, and incorporating the QTcIPA into a guided decision-support system, whereby healthcare providers can evaluate the risk of QTcIP and offer recommendations in a timely manner. This is particularly valuable in environments where access to expert consultations, such as cardiology services, is limited. The QTcIPA will be computerized into web-based and mobile applications with the goal of enhancing the assessment and management of QTcIP in settings which provide mental health care, both in hospitals and in the community.
